# Forced exercise activates the *NrF2* pathway in the striatum and ameliorates motor and behavioral manifestations of Parkinson's disease in rotenone-treated rats

**DOI:** 10.1186/s12993-020-00171-9

**Published:** 2020-11-06

**Authors:** Dina M. Monir, Motamed E. Mahmoud, Omyma G. Ahmed, Ibrahim F. Rehan, Amany Abdelrahman

**Affiliations:** 1 Department of Physiology, Faculty of Medicine, Sohag University, Sohag, 82524 Egypt; 2 Department of Animal Behavior and Husbandry (Genetics, Breeding, and Production), Faculty of Veterinary Medicine, Sohag University, Sohag, 82524 Egypt; 3 Department of Physiology, Faculty of Medicine, Assiut University, Assiut, 71526 Egypt; 4 Department of Husbandry and Development of Animal Wealth, Faculty of Veterinary Medicine, Menofia University, Shebin Alkom, Menofia, 32511 Egypt

**Keywords:** Parkinson, Rotenone, Behavioral tests, Exercise, *Nrf2. TFAM*, *Noq1*

## Abstract

**Background:**

Parkinson's disease (PD) is a common neurodegenerative disorder characterized by progressive loss of nigrostriatal dopaminergic neurons leading to dopamine depletion and problems of movement, emotions, and cognition. While the pathogenesis of PD is not clear, damage of dopaminergic neurons by oxygen-derived free radicals is considered an important contributing mechanism. This study aimed to evaluate the role of treadmill exercise in male Wister rats as a single treatment and as an aid-therapy with L-dopa for rotenone-induced PD. To study the role of the *Nrf2- ARE* pathway as a mechanism involved in exercise-associated improvement in rotenone-induced PD in rats.

**Method:**

Animals were divided into 5 groups, (Control, rotenone, rotenone\exercise, rotenone\L-dopa, and rotenone\exercise\L-dopa (combination)groups). After the PD induction, rats in the rotenone\exercise and combination groups were daily treadmill exercised for 4 weeks.

**Results:**

Treadmill exercise significantly improved behavioral and motor aspects of rotenone-induced PD. When treadmill exercise was introduced as a single intervention, it amended most behavioral aspects of PD, gait fully corrected, short-term memory, and motor coordination. Where L-dopa corrected locomotor activity and motor coordination but failed to improve short-term memory and only partially corrected the gait of rotenone-treated rats. When treadmill exercise was combined with L-dopa, all features of PD were corrected. It was found that exercise upregulated some of its associative genes to *Nrf2* pathways such as *TFAM, Nrf2* and *NQO.1* mRNA expression.

**Conclusion:**

This study suggests that forced exercise improved parkinsonian like features by activating the Nrf2 pathway.

## Background

Parkinson's disease (PD) is a common neurodegenerative disorder characterized by persistent loss of nigrostriatal dopaminergic neurons leading to depletion of dopamine and consequent problems of movement, emotions, and cognition. While many factors contribute to the pathogenesis of PD, damage of dopaminergic neurons by oxygen-derived free radicals is considered an important contributing mechanism [1, 2]. Rotenone is a potent inhibitor of complex I (NADH: ubiquinone oxidoreductase) of the mitochondrial electron transport chain. This allows for accumulation and overproduction of reactive oxygen species (ROS) which eventually leads to cell damage [3, 4]. Chronic rotenone exposure in rats causes both neuropathological findings and behavioral symptoms of PD [5]. The rotenone model mimics the gradual progression of PD as observed in humans. Systemic mitochondrial inhibition by rotenone leads to selective nigrostriatal degeneration [6].

Treadmill exercise is indicated as a physical therapy to improve motor symptoms in patients with PD. Exercise can improve and alleviate memory loss in elderly patients, and decrease the risk of developing PD. The neuroprotective potentials of exercise are great, but the underlying mechanisms remain a debatable issue. Evidence suggests that exercise neuroprotection is due to its neurotrophic effects, as exercise increases the availability of several neurotrophic factors [7]. Long-term exercise benefits brain functioning by increasing the blood and oxygen flow to the brain, mobilizing growth factors that promote neurogenesis and synaptic plasticity, releasing of neurotransmitters, such as dopamine (DA), noradrenaline, serotonin, and glutamate and consequently improve manifestations of the disease either at the motor or cognitive. levels in Alzheimer's animal model [8].

Exercise increases the antioxidant status in the striatum of animals and protects against neurological oxidative challenges. The nuclear factor erythroid-derived 2-like 2 (*Nrf2*)-antioxidant response element (*ARE*) signaling pathway, a major cellular defense mechanism against oxidative stress. Exercise activates *Nrf2* in human skeletal muscles and mouse heart [9]. The *Nrf2-ARE* signaling pathway appears to be a strong mechanism for exercise-induced neuroprotection [10]. The activation of the *Nrf2* gene activates genes that encode for antioxidant enzymes within the cells like *heme oxygenase* and *NADH quinone oxidoreductase (NQO1)*. Also, *Nrf2* activates the *mitochondrial transcription factor A (TFAM)* which regulates for mitochondrial DNA (mtDNA) replication [11]. Therefore, in this study, we investigated whether treadmill exercise as a single therapeutic intervention and as add-on therapy with L-dopa improve manifestations of PD in rotenone-treated rats. Also, we assessed the *Nrf2* pathway as a possible mechanism activated by treadmill exercise.

## Materials and methods

### Animals and experimental design

Fifty-adult male Wistar rats with body weight averaged 275 ± 25 gm, 9 months aged; were purchased from the Faculty of Science, Sohag University, and were housed in the Medical Animal Laboratory in Sohag Faculty of Medicine. Animals were allowed free access to food and tap water. Rats were housed in standard cages, at normal light/dark cycle and room temperature. The rats were randomly divided into 5 groups (n = 10 in each group); control group, rotenone-injected group, rotenone\exercise group, rotenone\L-dopa treated-group, and combined (rotenone\L-dopa\exercise) group. The study was approved by Research Ethics Committee considering the care and use of laboratory animals (permission number: SOH-IACUC-17050301).

### Induction of Parkinsonism

Rats were subcutaneously injected with either vehicle or rotenone (R8875; 95%, Sigma-Aldrich, USA) 2 mg/kg, for 4 weeks. Rotenone solution was prepared as a 50 × stock in 100% dimethylsulfoxide (DMSO) then in a medium-chain triglyceride, miglyol 812 N (Sigma) to obtain a final concentration of 2 mg/mL rotenone in 98% miglyol 812 N, 2% DMSO. The prepared solution was stored in an amber septa vial to be protected from light and inverted many times before each injection to eliminate the possibility of settling [12]. L-dopa (L-3, 4 dihydroxyphenylalanine methyl ester hydrochloride, (Sigma) was dissolved in normal saline and was administered at a dose 6 mg/kg/day, injected intraperitoneally for 4 weeks.

### Exercise protocol and treatments

After induction of PD, rats in the exercise group were forced to run in a 3-channel treadmill (Heath Life V4000M). The exercise regimen continues for 30 min/day, 5 times a week for 4 weeks. The treadmill speed accelerates beginning with 2 m/min during the first 5 min, at 3 m/min during the second 5 min, and then at 5 m/min for the last 20 min [13]. The efficiency of this exercise protocol was previously assessed by measuring the serum lactate dehydrogenase and creatine phosphokinase in a non-published experiment (Additional file 1: Fig. S1).

### Behavioral and motor analysis

These tests assess motor activity, and behavior of the rats, they were performed at the end of the experiment for all groups and included;

### Open field test (OFT)

OFT was performed according to the previously described method [14]. The apparatus was a squared plastic arena (114 × 114 × 44 height cm) its floor was divided into smaller squares (19 × 19 cm^2^) with a central square (38 × 38 cm^2^) each rat was put gently in the center of the arena and was left for 5 min moving and exploring the field. A video camera was fixed at the top of the arena to record the activity of the rat which was scored by a specialist who was blind to the experimental groups. Each animal was then given a score for total locomotor activity; calculated as the sum number of line crosses and rears, a score for exploratory behavior; the sum of the number of central square entries and the duration of time spent in the central square, and the anxiety score is equal to the sum of urination and defecation boli [14].

### Object recognition test (ORT)

ORT was performed as described by Walsh and Cummins [15]. To assess the short-term memory (STM) and long-term memory (LTM), four objects used were made of plastic material. The objects and arena were washed with a 10% ethanol solution after each trial. The training was conducted by placing each rat for 5 min into the open field apparatus, where two identical objects (objects A1 and A2) were put in two adjacent sides, 10 cm from the walls. In the STM test, a rat was given 1.5 h after training; the rats explored the open field for 5 min in the presence of one familiar (A) and one novel (B) object. LTM test was done 24 h after training, the same rat explored the field for 5 min in the presence of a familiar object A and a novel object C. Exploration is defined as sniffing or touching the object with the nose and/or forepaws. The time (T) which is spent by each rat exploring the different objects was measured as TA1, TB, and TC. These times were used for calculation of STM = [TB / (TA1 + TB)] 100, and LTM = [TC / (TA1 + TC)] 100.

### Foot print test

Footprint test was used to measure gait analysis by permitting rats to run in a wood corridor apparatus (65 × 5 × 15 cm^3^), which was lined with a pre-cut piece of white paper. Rats were trained to run to the end of the corridor by placing the rats at the far end of the corridor and encouraging them to move towards the end. The training was conducted twice for each rat until the animal could run to the end box without encouragement. For testing, the paws of the animal were painted with four non-toxic watercolors as described previously [16]. For each animal, the gait was calculated using 4 paw prints; this allowed 5 values-yield/rat; [front stride length (FSL), front stride width (FSW), hind stride length (HSL), hind stride width (HSW) and overlap (OL)] which were then averaged to provide gait measurements [15].

### Rota-rod test

To assess the motor coordination of the animals, we used an accelerating Rota-rod (Harvard Apparatus, UK). The Rota-rod consisted of a suspended rod, accelerating for 60 s, beginning from 5 rounds per minute (RPM) to reach 15 RPM, and continuing at that speed for a further 60 s. A trial was stopped when the rats fell off the Rota-rod or after they complete 120 s. The mean latency time of the three trials was taken. Animals were trained for five days to perform the test [17].

### Tissue Sampling

After behavioral tests, the rats were anesthetized with Zoletil (1 mg\kg i.p (Vibac Laboratories, Carros, France). Rats were transcardially perfused with 0.05 M phosphate-buffered saline (PBS). The brain was removed, separated into right and left hemispheres, snap-frozen in liquid nitrogen, and kept for 1 h at − 80 °C. The striatum was dissected through multiple manual coronal sections with a sharp razor blade and was collected with the help of two Dumont No. 5 forceps [18]. Samples then were stored in Eppendorf tubes at − 80 °C until further analyses.

#### (1) RNA extraction

Total RNA was extracted from 30 mg of tissue samples according to manufacture instructions (RNA Extraction kit (#K0731, Thermo Scientific, Lithuania)). They were extracted to measure Nrf2, NOQ.1, TFAM, and using housekeeping GAPDH by real-time polymerase chain reaction (real-time PCR).

#### (2) Reverse transcription

Extracted RNA concentration was quantified using Nanodrop spectrophotometry (Quawell 5000, USA); then 110 ng of total RNA transcribed using RNA reverse transcriptase kits ((#K0251) (Thermo Scientific, Lithuania)). The thermal cycler was programmed at 25 °C for 10 min, 37 °C for 120 min, 85 °C for 5 min, and 4 °C for 20 h.

#### (3) Real-time PCR

Prepared cDNA, was used in the qPCR analyzer (Step One, Applied Biosystems, Singapore) using the MAXIMA SYBR Green qPCR Master Mix with the following program: 1 cycle at 95 °C for 10 min; 40 cycles of 95 °C for 15 s, 60 °C for 30 s and 72 °C for 30 s; one cycle at 95 °C for 15 s, 60 °C for 1 min and 95 °C for 15 s. The specific primers (Table 1) *of Nrf2, TFAM,* NADPH dehydrogenase *(NQO1*) & housekeeping *GAPDH* were purchased from Metabion international AG, Germany. Fold expression (2^−ΔΔct^) was calculated according to the relative expression of housekeeping gene *GAPDH.*

**Table 1:** Forward and reverse primers for real time PCR

Gene name	Forward primer	Reverse primer	Access number	References
*NRF2*	5′ CACATCCAGACAGACACCAGT-3′	5′ CTACAAATGGGAATGTCTCTGC-3′	NM_031789	[19]
*TFAM*	5′AGTTCATACCTTCGATTTTC-3′	5′ TGACTTGGAGTTAGCTGC-3`	NM_031326.1	[20]
*NQO1*	5′CAGCGGCTCCATGTACT-3′	5′ GACCTGGAAGCCACAGAAG-3′	NM_017000	[19, 21]
*GAPDH*	5′CAGGCATATGGTGGTCCATAGAG-3′	5′ TCATGGGATCCACCTGCAGC-3′	NM_017008	[19]

*NRF2* nuclear factor erythroid 2 (NFE2)-related factor 2, *NQO1* quinone oxidoreductase 1, *TFAM* Mitochondrial transcriptional factor A, *GAPDH* glyceraldehyde-3-phosphate dehydrogenase

#### (4) Tyrosine hydroxylase

Homogenized striatum of the right hemisphere of all rat brains was used to measure the levels of tyrosine hydroxylase enzyme by ELISA (tyrosine hydroxylase (TH) rat ELISA kits (#:96,791) from Glory Science Co., (Ltd, China) with a detection range 0.625- 20 ng\ ml.

### Statistical analysis

Statistical package for social sciences (IBM-SPSS), version 24 IBM- Chicago, USA (May 2016) was used for statistical data analysis. Data expressed as mean ± standard deviation (SD), number, and percentage. A one-way analysis of variance (ANOVA) test was used to compare the means of the analyzed groups. Post hoc test (LSD type) was used for multiple comparisons between each group and the other. P-value was considered significant when P < 0.05.

## Results

### Treadmill exercise, L-dopa and their combination improved exploration, and locomotion in rotenone-treated groups (Fig. 1)

**Fig. 1:**
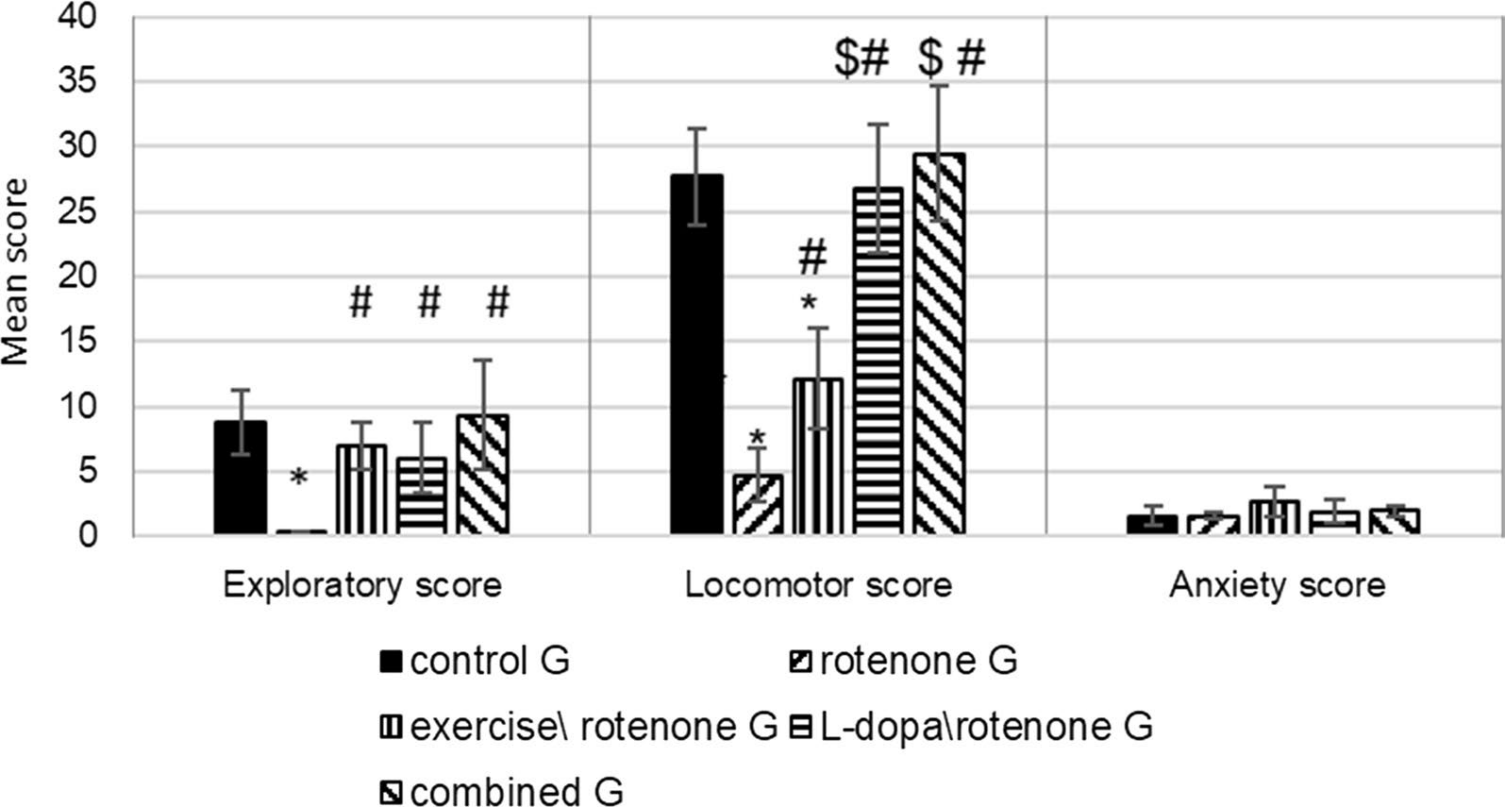
Open field test effect of exercise (4 weeks treadmill), L-dopa (6 mg\kg IP), and their combination on rotenone treated rats (2 mg\kg SC for 4 weeks). N = 10 rats\group. The analysis was done by ANOVA test. P-value < 0.05, *compare to control G, # in comparison with the rotenone G

After 4 weeks of daily administration of rotenone, rats spent less time in locomotion and exploration of open field environment compared to the vehicle-injected group (p = 0.00007, Fig. 1). But the anxiety scores in terms of urination and defecation times were not different between both groups. To sum, rotenone injection decreased exploration and locomotion scores in the open field test.

It was noticed that after injection of rotenone, rats practiced 5 times/week a treadmill exercise spent less time in the central arena and increased number of line crossings in the open-field test compared to the non-exercised rotenone group (p = 0.0004). Similarly, after rotenone-administration, in rats treated with L-dopa and L-dopa co-treatment with exercise practice recovered the locomotor and exploratory activities after rotenone treatment. However, L-dopa\rotenone and the combined exercise\L-dopa\rotenone groups showed significantly higher locomotor scores than the exercise\rotenone group, (p = 0.0009). There was an insignificant difference regarding exploration (p = 0.50) in-between the three treatment groups. Also, there was no significant change concerning the anxiety score between the five groups under the study.

### Treadmill exercise alone and in combination with L-dopa improved STM in the rotenone-treated groups (Fig. 2)

**Fig. 2:**
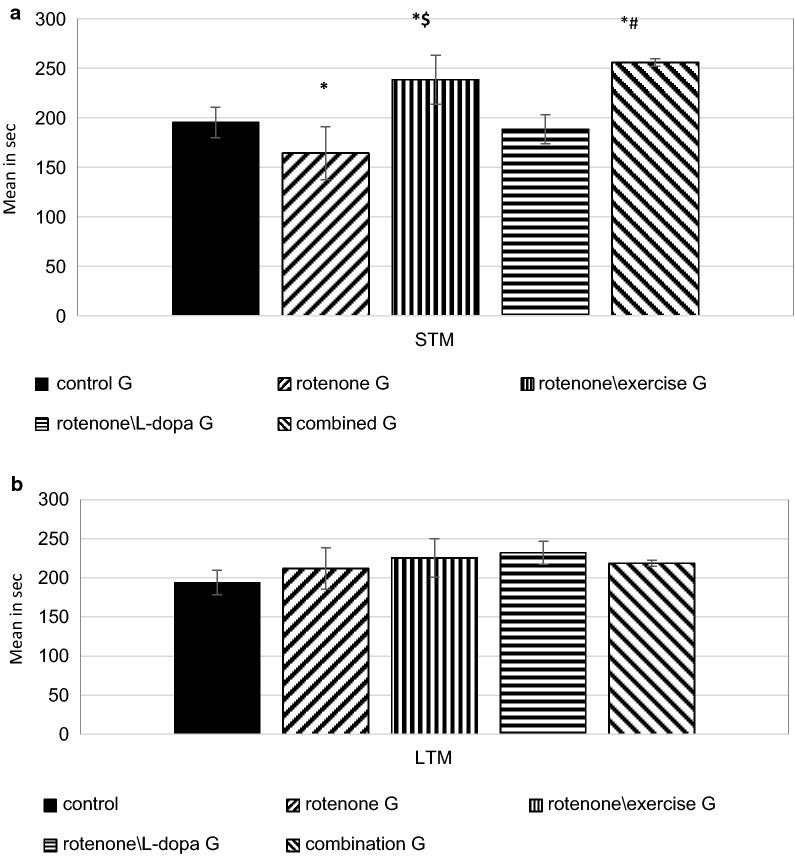
Object recognition test, the effect of exercise (4 weeks treadmill), L-dopa (6 mg\kg IP), and their combination on rotenone treated rats (2 mg\kg SC for 4 weeks) on STM (Fig a) and LTM (Fig b). The analysis was done by ANOVA test. P-value is considered significant when P < 0.05, n = 10 rat in each group. *compare to control group, ^#^in comparison with the rotenone treated group. *STM* short term memory, *LTM* long term memory

There was a significant decrease in STM of the rotenone-treated group compared to the control group (p = 0.02). No significant difference as regard LTM in either group.

L-dopa alone didn't improve STM in the rotenone treated rats (p = 0.23) while, exercise alone or its combination with L-dopa has produced significant improvements in the STM in comparison with the rotenone-injected group (p = 0.03). Meanwhile, LTM was insignificantly changed among all groups (p = 0.38).

### Treadmill exercise alone and in combination with L-dopa fully corrected the gait, while L-dopa alone caused a partial correction of gait analysis

Rotenone injection caused a significant gait impairment in comparison to the control rats in terms of increased overlap (OL) distance (p = 0.001), shortened both stride length; hind (HSL) and front (FSL) steps, and similarly significant wide base, as detected by increased FSW and HSW (p = 0.03, Table 2).

**Table 2:** Effects of exercise, L-dopa and their combination on gait measured by Foot print test (overlap, front stride width, front stride length, hind stride width and hind stride length in cm)

Group	Control G	Rotenone G	Rotenone\ exercise G	Rotenone\L-dopa G	Combined G	P- value by ANOVA
OL	1.50 ± 0.3	2.10 ± 0.45	1.40 ± 0.3	1.35 ± 0.2	1.55 ± 0.3	0.0008
		*P = 0.001	^#^P = 0.001	^#^P = 0.0001	^#^P = 0.003	
FSW	4.95 ± 1.23	5.95 ± 0.83	3.40 ± 0.99	5.20 ± 0.91	4.15 ± 0.8	0.006
		*P = 0.02	^#^P = 0.007	P = 0.12	^#^P = 0.009	
FSL	11.89 ± 1.8	9.70 ± 1.65	13.5 ± 3.2	13.1 ± 1.44	11.1 ± 2.5	0.002
		*P = 0.01	^#^P = 0.001	^#^P = 0.006	^#^P = 0.003	
HSW	6.15 ± 0.78	7.00 ± 0.88	4.55 ± 1.2	6.15 ± 0.78	5.65 ± 0.7	0.001
		*P = 0.004	^#^P < = 0.001	P = 0.08	^#^P = 0.002	
HSL	12.65 ± 1.6	10.1 ± 1.46	12.8 ± 1.53	13.5 ± 1.24	11.7 ± 2.5	0.001
		*P= 0.02	^#^P=0.002	^#^P=0.001	^#^P=0.03	

P value is calculated by ANOVA test, and is considered significant if < 0.05. n = 10 in each group. Data was expressed as mean ± SD

*OL* overlap, *FSW* front stride width, *FSL* front stride length, *HSW* hind stride width, *HSL* hind stride length

*significant when compared to control group. ^#^significant when compared to rotenone group

Treadmill exercise alone and exercise /L-dopa treatment corrected OL distance induced by rotenone injection (p = 0.001). Additionally, increased the FSL and HSL (p = 0.0009), and decreased the HSW and FSW (p = 0.0008). L-dopa treatment improved the asymmetrical gait and caused an elongation in the stride length, and a decrease in OL in the rotenone-injected group (p = 0.001), while it did not affect HSW (p = 0.08) and FSW (p = 0.12). To sum, treadmill exercise alone and in combination with L-dopa treatment fully corrected the gait of rotenone-injected rats.

### Treadmill exercise, L-dopa, and their combination prolonged the latency time to fall in the Rota-rod test (Fig. 3)

**Fig. 3:**
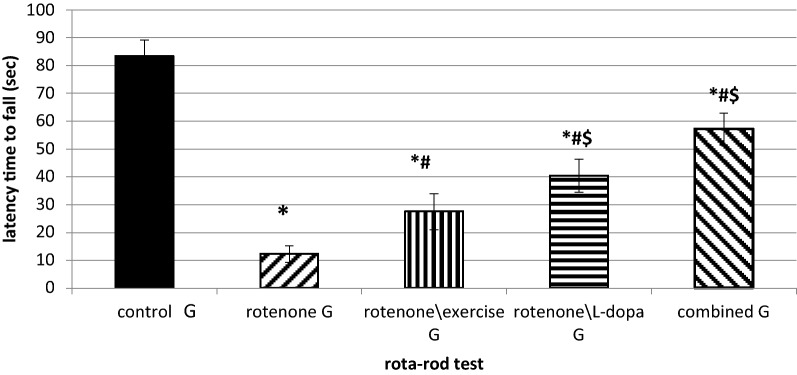
Rota-rod test, the effect of exercise (4 weeks treadmill), L-dopa (6 mg\kg IP daily for 4 weeks), and their combination on rotenone treated rats (2 mg\kg SC for 4 weeks). P-value was calculated by ANOVA test and was considered significant if < 0.05. n = 10 in each group. *significant when compared to the control group. ^#^significant when compared to the rotenone-treated group. ^$^Significant when compared to rotenone\exercise G

There was a significant decrease in latency time to fall after 4 weeks of a daily injection of rotenone in rats compared to the control group (p = 0.00008). Rats in all groups exhibited significant increases in the latency time to fall, in comparison to the rotenone-injected group (p = 0.001). However, the L-dopa and the exercise/L-dopa-treated group showed a longer latency time than the exercise\rotenone group (p = 0.02). Therefore, forced exercise, L-dopa treatment, and their combination prolonged the latency to fall in the Rota-rod test (Fig. 3).

### Effects of treadmill exercise, L-dopa and their combination on tyrosine hydroxylase (TH) levels in the striatum

**Fig. 4:**
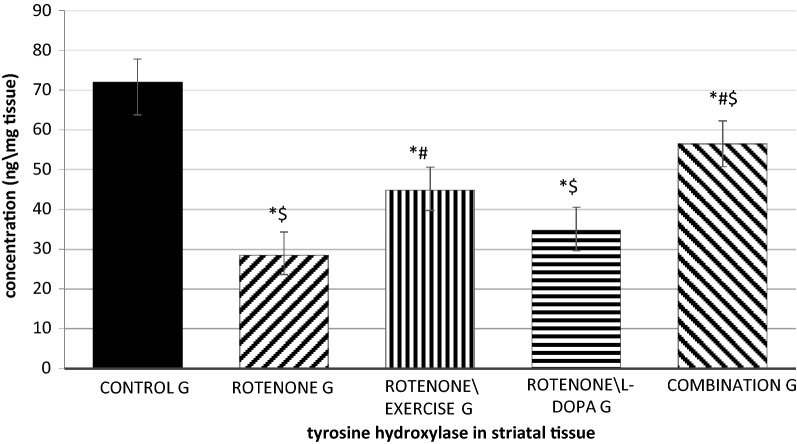
Striatal tyrosine hydroxylase measured by ELISA, effect of exercise (4 weeks treadmill), L-dopa (6 mg\kg, IP daily for 4 weeks), and their combination on tyrosine hydroxylase level in rotenone-treated rats (4 weeks, 2 mg\kg, SC). P-value is obtained by ANOVA and is considered significant if < 0.05. *significant in comparison to the control group, ^#^significant when compared to the rotenone group, ^$^significant when compared to the rotenone exercise group. n = 10 in each group

Rotenone administration in rats significantly reduced TH levels (to 40% of the control value) in the corpus striatum when compared to the vehicle-injected group (P = 0.00006). Whereas, forced exercise and their combined applications caused a significant increase in striatum TH levels which increased up to 62% and 78% of normal) when compared to the rotenone-injected group (P = 0.001). L-dopa alone didn't significantly increase the striatal TH level when compared to the rotenone group (P = 0.17). Besides, the tyrosine hydroxylase level in the L-dopa\rotenone group was significantly lower than that of the exercise\ rotenone and the combined group (P = 0.09, Fig. 4).

### Effects of treadmill exercise, L-dopa and their combination on Nrf2 expression in the striatum

Rotenone caused a significant increase of *Nrf2 mRNA* expression in the striatum of rats in comparison to the vehicle-injected group (P = 0.02).

**Fig. 5:**
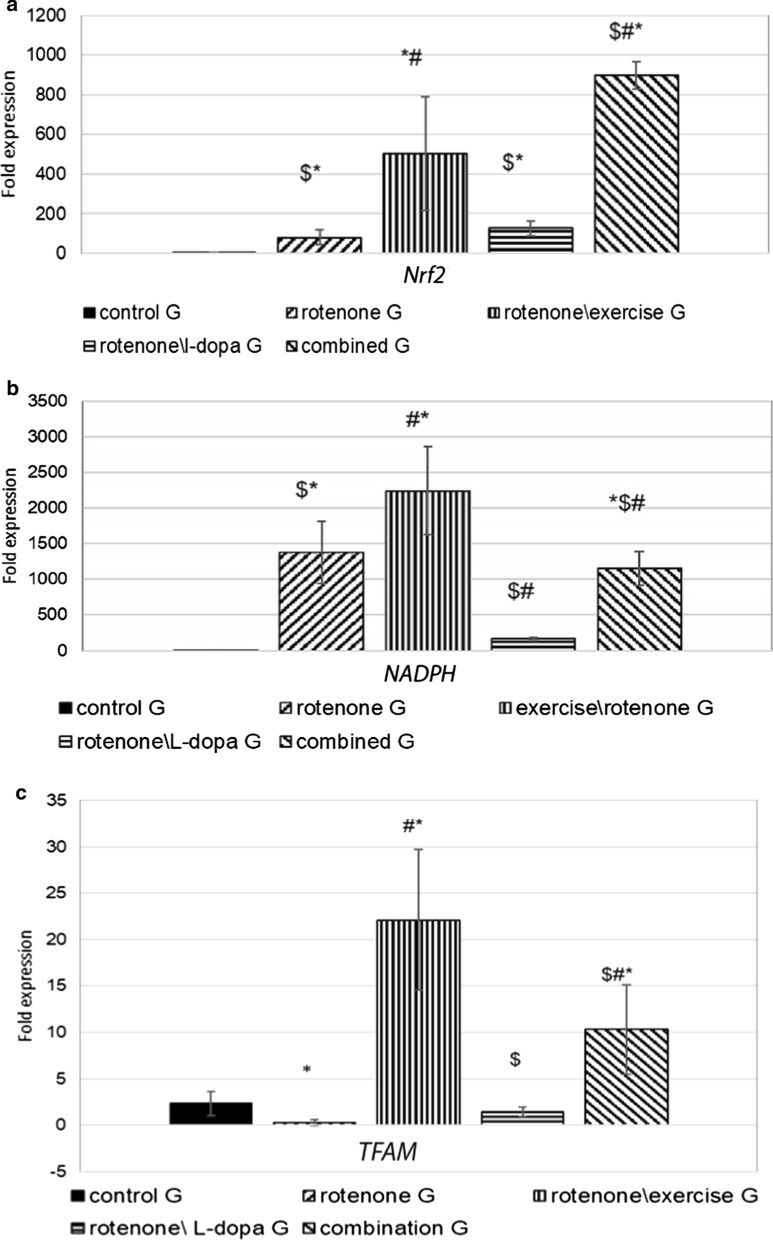
Expression levels of genes by PCR in the corpus striatum, effect of exercise (4 weeks treadmill), L-dopa (6 mg\kg IP daily for 4 weeks), and their combination on rotenone treated rats (2 mg\kg SC for 4 weeks). **a** *Nrf2* gene, **b** expression level of NADPH dehydrogenase *(NQO1)* mRNA, and **c** expression level of *TFAM* mRNA, P-value is calculated by ANOVA test, and is considered significant if  < 0.05. n = 10 rats in each group. *Significant when compared to control G. ^#^significant when compared to the rotenone group. ^$^Significant when compared to the rotenone\exercise group

Forced exercise alone and in combination upregulated *Nrf2 mRNA* expression in corpus striatum when compared to the rotenone-injected group (P = 0.0009). However, treatment with L-dopa did not affect *Nrf2 mRNA* expression (P = 0.62). Moreover, *Nrf2 mRNA* expression was lower in the L-dopa\rotenone group compared to the exercise\rotenone group (P = 0.00). Interestingly, the combination between forced exercised and L-dopa treatment showed a significant increase in *Nrf2 mRNA* expression in comparison to the exercise\rotenone group (P = 0.03) and L-dopa\rotenone group (P = 0.001). This study showed that the administration of rotenone increased *Nrf2* expression in the corpus striatum, such upregulation was augmented by forced exercise, dramatically increased by the combination of treatment of L-dopa and forced exercise, but was not affected by single treatment of L-dopa (Fig. 5a).

### Effects of treadmill exercise, L-dopa and their combination on target genes of Nrf2 in the striatum

Rotenone significantly increased expression of *NQO1* in the rotenone group compared to control (P = 0.0009) as shown in Fig. 5b. Exercise produced a significant increase in *NQO1* mRNA expression level when compared to the rotenone group (P = 0.001). However, the L-dopa treatment reduced *NQO1* mRNA expression in the rotenone-injected group (P = 0.001). Whereas when exercise performed with L-dopa treatment did not change *NQO1 mRNA* expression compared to the rotenone group (P = 0.49), but decreased *NQO1 mRNA* expression when compared to the exercise\rotenone group (P = 0.003). Additionally, there was a significant increase in *NQO1* expression in the exercise\L-dopa\rotenone group compared to the L-dopa\rotenone group (P = 0.001) (Fig. 5b).

Consistently, treadmill exercise caused a significant increase in TFAM mRNA expression after injection of rotenone and when practiced in combination with L-dopa treatment compared to rotenone group (P < 0.001) and L-dopa\rotenone (P = 0.0009 and = 0.01, successively). Similar to *NQO1* expression, L-dopa treatment did not affect *TFAM mRNA* expression in the rotenone-injected group (P = 0.73). There was a significant increase in *TFAM mRNA* expression in the combination group when compared to the exercise\rotenone group (P = 0.05), (Fig. 5c).

Overall, our results demonstrated that 4 weeks of daily treatment with rotenone increased Nrf2-related genes, *NQO1* and *TFAM*, expression in the corpus striatum. Such expression was enhanced by forced exercise and was not affected by a single treatment of L-dopa (Fig. 5b, c).

## Discussion

Parkinson's disease (PD) is a common neurodegenerative disease, about 1% above the age of 65 years suffer from. In PD, there is a progressive degeneration of dopaminergic neurons of the substantia nigra of the midbrain [22]. This results in the characteristic motor impairment and the extra motor manifestation of the disease. Rotenone is known to induce PD in rats by targeting dopaminergic neurons [23]. It acts by inhibiting complex I of the mitochondrial electron transport chain and causing accumulation of reactive oxygen species (ROS) and subsequently, cell damage. Age-related mitochondrial dysfunction and oxidative stress have been strongly involved in the pathophysiology of PD [24, 25].

In this study, rotenone 2 mg\kg\day was injected by the subcutaneous route for 4 weeks to induce rotenone-Parkinson's disease rat model. The rotenone-treated group, showed a significant decrease in exploration and locomotion as regards to the open field test, gait impairment; (asymmetrical foot pattern, shortened stride length, and widened base), a significant decline in motor coordination was observed by shortened latency time on Rota-rod. Cognitive function impairment of PD also existed; as a significant decrease in novel object preference in the short-term memory test. Tyrosine hydroxylase levels in the striatum significantly decreased in rotenone treated rats. Similar to our study, Vijayalakshmi et al. [26] and Valdez et al. [27] reported a significant decrease in locomotion in the Open field test in rotenone-treated Wister rats. von Wrangel et al. [28], and Cannon et al. [12] reported that rotenone-treated rats showed significant impairment in Rota-rod and significant decline in tyrosine hydroxylase in the striatum. As regard footprint test, the results of this study were in line with Madiha et al. [29] their data showed significantly impaired walking pattern and shortened stride length in rotenone-treated rats.

Treadmill exercise significantly increased exploration and locomotion in rotenone-treated rats. Treadmill exercise corrected the gait impairment in rotenone-treated rats; the asymmetrical patterns were corrected by a significant reduction in overlap, accompanied by a significant increase in stride length and a significant decrease in stride width. Treadmill exercise significantly improved short-term memory novel object recognition in the rotenone-treated group. Tyrosine hydroxylase levels significantly increased in the brain after treadmill exercise. Exercise also improved motor coordination as it increased latency time to fall on Rota-rod significantly. The beneficial effects of the treadmill and other means of exercise in improving motor coordination (prolong latency time to fall on Rota-rod) were also reported by Shin et al. and Lee et al. [30, 31], in the rotenone model of PD. In line with our study, Chen et al. [32] reported a significant improvement of the Rota-rod test in addition to a significant gait improvement (improved overlap, stride length, and base width) with treadmill exercise but in 6-OHDA model PD.

When L-dopa was administrated in rotenone-treated rats, a significant improvement regarding exploration and locomotion was found. Motor co-ordination significantly improved ever more than exercise alone. The gait showed significant partial improvement, the asymmetrical pattern was significantly corrected, and the stride length significantly increased, meanwhile, short-term memory did not significantly improve. Tyrosine hydroxylase levels did not significantly increase in the brain. Our results agree with Allam et al. [33] who found a significant increase in locomotor activity and exploration with L-dopa treatment of rotenone-treated rats in the open field test, and Sgroi et al. [34] who reported a significant increase in locomotion and motor co-ordination with 8 mg\kg L-dopa treatment for ten days in 6-OHDA model of PD.

When L-dopa treatment was accompanied by treadmill exercise, there was a significant increase in exploration and locomotion behavior in the open field. A significant increase in the latency time of the Rota-rod test was found indicating improved motor coordination. There was a significant gait improvement with L-dopa and exercise co-treatment, the asymmetrical pattern was fully corrected, with a significant increase in stride length and a significant decrease in stride width. Short-term memory significantly improved. Tyrosine hydroxylase levels in the brain were significantly increased.

In our study, we investigated some genes of the *Nrf2-ARE* pathway as a possible mechanism involved in the treadmill exercise effects on the brain. Exercise causes an increase in O2 consumption with an increase in ROS production, especially H2O2. Oxidative stress leads to inhibition of *KEAP-1*, dissociation of *Nrf2* from *KEAP-1* in the cytoplasm, migrates into the nucleus, and activates the *Nrf2-ARE* [35]. *Nrf2* activates anti-oxidant gene expression with increased productions of detoxifying enzymes: *Heme oxygenase*, and *NADPH quinone oxidoreductase (NQO.1*). Activation of *Nrf2* together with *PCG1α*, which also is activated by exercise, caused an increase in *mitochondrial transcription factor A (TFAM)* expression in the striatum. *TFAM* is a nuclear factor that controls the replication of mitochondrial DNA (mt DNA). The upregulation of *TFAM* increases the number of copies and packaging of mtDNA. Also, adequate levels of TFAM are required to prevent mtDNA release into the cytoplasm and initiate an inflammatory response [11].

This study measured the rate of expression of *Nrf2, TFAM, and Nqo.1* in the striatum of rotenone-treated rats. Rotenone downregulated expression of the *TFAM* in the rotenone group while upregulated *Nrf2* and *Nqo1* in comparison to the control rats. Treadmill exercise showed a significant increase in *Nrf2, TFAM*, and *NQO.1* in the striatum in the exercise\rotenone group in comparison to control and rotenone groups. L-dopa\rotenone group showed an insignificant change in the expression of these genes. However, the exercised \L-dopa\rotenone group, showed a significant increase in *Nrf2, TFAM*, and *Nqo.1* expression, when compared to both control and rotenone group.

Whether rotenone increases or decreases the expression of *Nrf2* and *NQO1* is a matter of debate. Many studies [36] found that rotenone in in-vivo and in-vitro studies downregulated the expression of these genes and enhanced apoptosis of the neurons in the nigrostriatal tissue which was reversed by administration of danshensu herbal extract. Similarly, Cui et al. [37] found decreased expression of NRF2 and NQO1 proteins measured by western blotting in rotenone treated rats which were corrected with pretreatment with curcumin. On the contrary, Wei et al. [38] found that rotenone increased *NRF2* and *NQO1* expression in the striatum which was further enhanced by ellagic acid. They measured the NRF2 protein in the cytoplasm and in the nucleus of the cell indicating that the expression of these transcription factors was increased in the nucleus.

As regard expression of *TFAM*, this is the first study which investigated its expression in rotenone-treated rats, Rotenone downregulated *TFAM* expression which may underlie the instability in the mtDNA and consequent damage of the dopaminergic neuron. This is evidenced by the decreased level of tyrosine hydroxylase measured in the striatum. Noteworthy, Chen et al. [39] in their study on post mortem human parkinsonian patients found that expression of *TFAM* in substantia nigra is lower and their mtDNA is less stable in comparison to age-matched elderly control subjects.

As regards our study results, treadmill exercise activated the *Nrf2* pathway in the striatum of the rotenone-treated rat. This activation could be one of the mechanisms involved in treadmill exercise beneficial effects on the rotenone-treated model of PD. Few studies which measured *TFAM* expression in animal models e.g. Aguiar et al. [40] reported that exercise significantly increased *Nrf2* and *TFAM* in the striatum of 6-OHDA hemiparkinsonism. Other studies reported *TFAM* activation with exercise in different pathological conditions other than parkinsonism. For instance, Lashgarie et al. [11] reported that treadmill exercise increased *TFAM* expression in the heart of nicotine sensitized rats, which attenuated mitochondrial-mediated damage in the myocardium induced by nicotine.

## Conclusion

In this study, treadmill exercise increased tyrosine hydroxylase and activated the *Nrf2* pathway and some of its associated genes however L-dopa could not. Where L-dopa corrected locomotion, exploration, and motor co-ordination but failed to improve short-term memory and only partially corrected the gait of rotenone-treated rats. When treadmill exercise was combined with L-dopa, all the behavioral motor and non-motor aspects of PD were corrected.

## Recommendation

Exercise is highly recommended and is a mandatory approach for the treatment of parkinsonism in humans. Further studies are needed to explore the importance of expression of the *TFAM* gene as part of the *Nrf2* pathway in protecting against mitochondrial induced oxidative damage.

## Supplementary information

**Additional file1: Figure S1.** The effects of exercise on serum levels of CPK and LDH in control groups, * if the differences were significantly different from the control group.

## Data Availability

The datasets used and/or analyzed during the current study are available from the corresponding author on request.

## Abbreviations

ARE: Antioxidant response elements
DA: Dopamine
*NQO1*: NAD(P) H quinone oxidoreductase 1: NAD(P)H dehydrogenase
*Nrf2*: Nuclear factor erythroid 2 (NFE2)-related factor 2
PA: Physical activity
*PGC1α*: Peroxisome-proliferator-activated receptor-γ coactivator-1α
ROS: Reactive oxygen species
*TFAM*: Mitochondrial transcriptional factor A
OL: Overlap
FSL: Forward stride length
FSW: Forward stride width
HSL: Hind stride length
HSW: Hind stride width
