# Supplementary figures and images for: Forced exercise activates the NrF2 pathway in the striatum and ameliorates motor and behavioral manifestations of Parkinson's disease in rotenone-treated rats
Source: Behav Brain Funct. 2020 Nov 6;16:9. doi: 10.1186/s12993-020-00171-9 (PMC7646065; doi:10.1186/s12993-020-00171-9)

## Slide 1
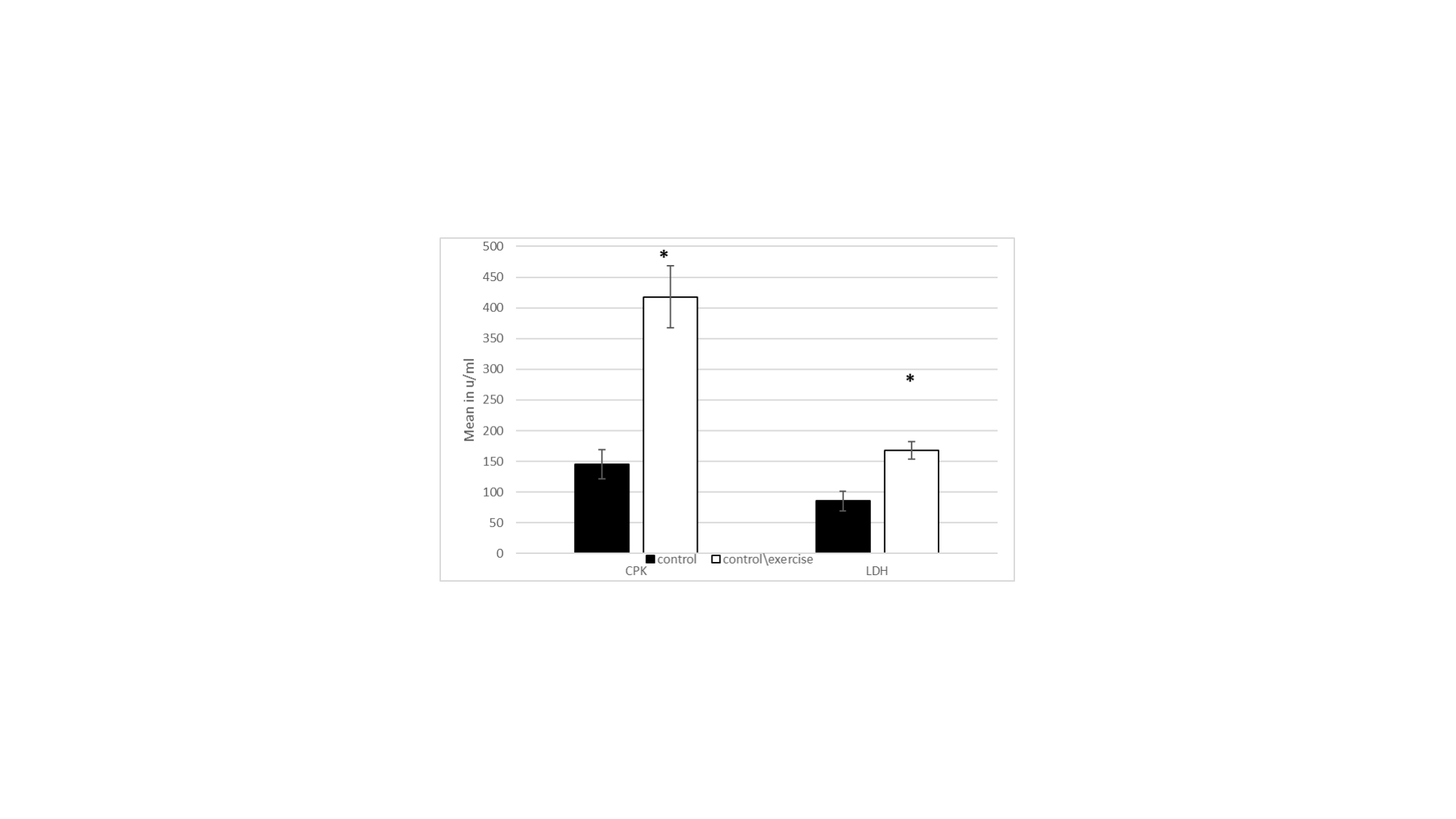

Supplement: Supplementary file 1 — Additional file1: Figure S1. The effects of exercise on serum levels of CPK and LDH in control groups, * if the differences were significantly different from the control group. [file 12993_2020_171_MOESM1_ESM.pptx]
